# Current trends in health coaching for chronic conditions: A systematic review and meta-analysis of randomized controlled trials

**DOI:** 10.1097/MD.0000000000021080

**Published:** 2020-07-24

**Authors:** Juan Yang, Brent A. Bauer, Stephanie A. Lindeen, Adam I. Perlman, Abd Moain Abu Dabrh, Kasey R. Boehmer, Manisha Salinas, Susanne M. Cutshall

**Affiliations:** a Division of General Internal Medicine, Mayo Clinic, Rochester, Minnesota, United States; b Department of Pain Medicine, Shenzhen Nanshan People's Hospital, Shenzhen, Guangdong, China; c Division of General Internal Medicine; d Division of Family Medicine, Mayo Clinic, Jacksonville, Florida; e Division of Knowledge and Evaluation Research, Mayo Clinic, Rochester; f Division of General Internal Medicine, Mayo Clinic, Jacksonville, Florida, Minnesota, United States.

**Keywords:** chronic condition, health coaching, randomized controlled trial, review

## Abstract

**Background:**

Chronic conditions are placing a serious burden on individuals as well as the health care system. Health coaching (HC) has emerged as a promising approach that can support effective lifestyle interventions for chronic conditions. However, until now there is no particularly comprehensive systematic review of HC impact on a chronic condition from the angle of patient improvement and detail coaching characteristics reported.

**Objective:**

To synthesize available studies on the efficacy and current status of HC interventions on the health of chronically ill adult patients.

**Methods:**

The literature search will be conducted for trials published in English within the past four years. Electronic databases CINAHL, Cochrane Library, Embase, MEDLINE, and Scopus will be searched with keywords describing HC for chronic diseases. Randomized controlled trials that compare HC interventions to conventional care or other alternative therapies will be included. Data extraction will be conducted by two reviewers independently, and enrolled trials will be evaluated for quality and bias assessment. If appropriate, meta-analysis will be conducted on the last stage of the review; otherwise, the study findings will be described narratively. The software Review Manager (Revman version 5.3.5.) provided by the Cochrane Collaboration will be applied for the meta-analysis.

**Results:**

This is the first study to comprehensively explore the effectiveness and current status of HC intervention for patients with chronic conditions.

**Discussion:**

Study findings from this review will advance the appropriate utilization of coaching practice by determining whether HC is effective and feasible among patients with chronic disease. If proven effective, this approach may be applied more broadly through public health interventions. The current status findings will also provide evidence to inform decisions for integrating HC interventions into the current management pathway for individuals with chronic conditions.

**Systematic review registration:**

PROSPERO CRD42020153280.

## 1 Introduction

A chronic condition, also known as “chronic disease” and “chronic illness”, which defined by the Centers for Disease Control and Prevention refers to a disorder that lasts no less than 1 year and requires ongoing medical management or limits activities of daily living or both.^[1]^ The most common chronic conditions include asthma, cancer, cardiovascular disease(CVD), diabetes, mental illness, etc.^[2]^ Statistics indicate that at least half of the population over age 65 has one or more chronic conditions and the rate is rising.^[3]^ Chronic conditions have been posing a growing public health problem throughout the world. In the United States of America, about 60% of the population lives with at least one chronic condition, while 42% with multiple ones. Among the top 10 causes of death, 7 are chronic conditions, especially both cancer and heart disease account for approximately half of all deaths annually.^[4]^ Chronic conditions have been the nation's major cause of death and disability.^[5]^ Patients with chronic conditions account for $3.3 trillion in annual health care costs^[6]^ and the majority of visits in primary care.^[7]^ Chronic conditions not only reduce the patients’ quality of life, but they also place a significant social and economic burden on individuals and health care systems.

As medical cares have become more complex, patients and medical providers exploring methods to address the chronic conditions increasing. Chronic conditions are partially preventable or modifiable by enhancing patients’ self-management. Health coaches have been at increasing use to assist patients with managing chronic conditions and to work toward lifestyle and behavior changes. Health coaching (HC), which was defined by Huffman & Miller in 2015, refers to the delivery of patient-centered care for their better wellness, improved health, lowered risk, as well as decreased costs.^[8]^ HC is patient-oriented and is used to motivate individuals to adopt health care interventions to help them enhance the quality of life and improve health.

However, previous reviews about the impact of HC interventions on chronic conditions vary. A rapid review published in 2013 targeted telephone-based coaching which includes two-way conversations by telephone or video phone between patients and providers. Results showed that telephone coaching services could improve health behavior, health status and self-efficacy of patients with one or more chronic diseases. This was, especially true for vulnerable people who had difficulty accessing health services.^[9]^ Kivela et al^[10]^ made a systematic review of the effects of HC on an adult with chronic conditions in 2014. They found HC could improve behavioral, physiological, social, and psychological outcomes. However, future research regarding long-term efficacy was recommended. A review protocol in 2016 planned to evaluate the effects of HC on individuals with chronic conditions. This review protocol included randomized controlled trials (RCT) or quasi-experimental studies published from February 2006 to February 2016, the research results of the formal review have been expected all the time.^[11]^ Kelly et al^[12]^ evaluated the efficacy of telehealth method to deliver the diet interventions in chronic disease patients for overcoming patient-centered limitations and study results showed telehealth diet interventions improve nutrient focus. It also highlighted the great importance of delivering complex dietary interventions in future researches. Pirbaglou et al^[13]^ assessed the impact of personal HC on type 2 diabetes and found that it was effective in glycemic self-management and advocated more research into the efficacy of each program component. A scoping review on lifestyle coaching for mental health difficulties was made in 2018, Study results indicated lifestyle coaching posed an extensive potential, but further research and practice where needed.^[14]^ McBrien et al^[15]^ summarized the evidence of patient navigator programs in patients with chronic diseases. Their findings indicated that those programs improved care processes, compared to usual care. In the most recent review, Boehmer et al^[16]^ provided a summary of health and wellness coaching activities in the field of multi-morbidity and called for a new type of intervention, capacity coaching in 2019. Currently, there is no comprehensive systematic review of HC impact on a chronic condition from the angle of patient improvement and detail coaching characteristics, therefore, a systematic review of randomized clinical trials from a comprehensive angle assessing the effect of HC in people with chronic conditions is needed. Overview of previous literature reviews investigating the impacts of Heath coaching on chronic conditions is seen in Table 1.

**Table 1:**
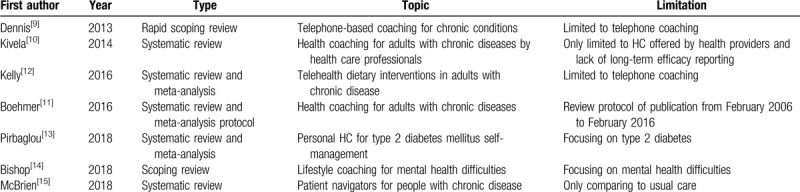
Overview of previous literature reviews investigating the impacts of Heath coaching on chronic conditions.

In this study, we plan to perform a systematic review with more evidence from previous published RCTs to evaluate the effects and current status of HC interventions for patients with chronic conditions with a very broad view.

The proposed systematic review will address the following two research questions:

1. Does HC benefit the physiological, biomarker and behavioral aspects status of chronic conditions patients, in comparison to the conventional clinical cares or alternative interventions?
2. What are the detail characteristics of current HC interventions for patients with chronic conditions, regarding their model, effect, techniques, sessions, duration of session prescribed, and coaching providers?

## 2 Methods

### 2.1. Aims

We aim to explore the effects and current status of HC on adult patients with chronic disease by reviewing the literature published from March 2016 to February 2020.

### 2.2. Study eligibility

Specific Inclusion/Exclusion Criteria are performed using the Population, Interventions, Comparators, Outcomes, and Study designs (PICOS) framework (Summarized in Table 2).^[17]^

**Table 2:**
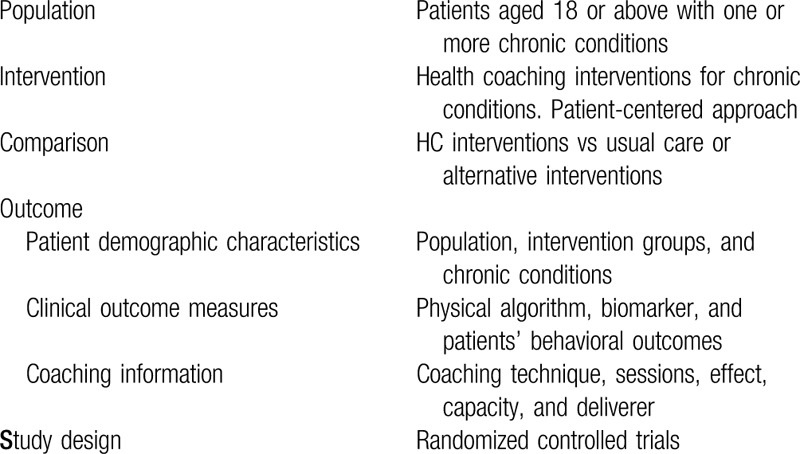
Outline of PICOS components.

#### 2.2.1 Types of participants

Studies that include patients aged 18 or above with one or more chronic conditions are included in this review. Chronic conditions, defined by the Centers for Disease Control and Prevention will be applied in this review.^[1]^ Although chronic conditions cover a diverse group of diseases, the most common conditions include, but not limited to the most common chronic conditions such as asthma, cancer, CVD, diabetes, mental illness and so on.^[2]^

#### 2.2.2 Types of interventions

Studies that deliver HC interventions for chronic conditions will be included. HC is defined as a patient-centered approach, guided by a coach, which assists patients to build their goals, learning skills and education toward their goals, as well as self-monitoring of behaviors to increase accountability.^[18]^ Techniques implemented by trained staff, health professional providers, or peer coaches will be considered.

#### 2.2.3 Types of comparators

Any study compares HC interventions with conventional care or alternative therapy will be considered for inclusion in this review.

#### 2.2.4 Types of outcomes

The outcomes for this review will consider HC relevant measurements which include, but not limited to the following outcomes:

1. Demographic characteristics
  Participants’ demographic information will be measured, including population, chronic conditions and intervention groups.
2. Clinical outcomes
  The following three types of clinical outcome measures will be evaluated by two independent reviewers in this review, including physical disease-specific outcomes, and patients’ behavioral outcomes.
3. Coaching information
  Coaching details will be assessed including coaching technique, sessions, effect, compactly, and deliverer.

#### 2.2.5 Type of studies

RCTs published in English, designed to compare HC to conventional cares or other alternative therapies are included. Chronic conditions, patient-centered trials are considered, while those trials applied in a wellness setting just for chronic condition prevention will be excluded, for example coaching applied in the workplace or Gym for health keeping or at work performance improvement, etc.

#### 2.2.6 Exclusion criteria

Studies where eligibility criteria are not clearly defined, duplicate publications and papers published beyond three years period or in any other language than English. Reasons for study exclusion will be kept in a file.

### 2.3. Search strategy

**Figure 1:**
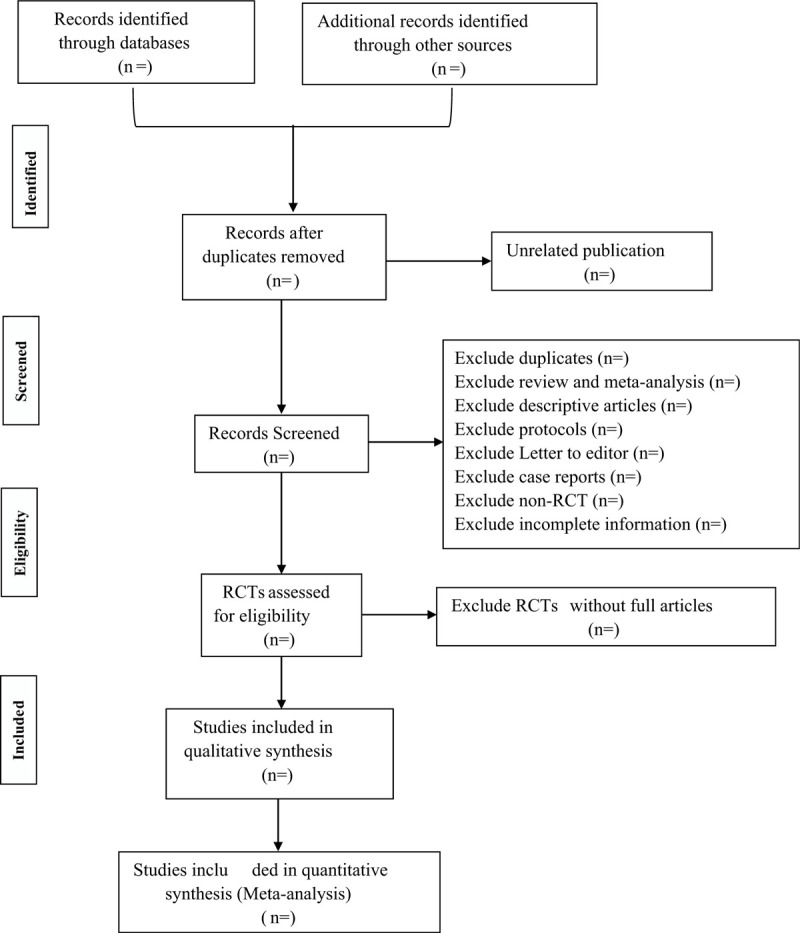
PRISMA flow chart of study selection.

This data search is designed and conducted by an experienced Mayo Clinic librarian with input from the study's principal investigator. This comprehensive literature search will be performed to identify publications in the following electronic databases such as CINAHL, Embase, Cochrane Library, MEDLINE, and Scopus from March 2016 to February 2020. Controlled vocabulary supplemented with keywords was used to search for studies describing HC for chronic diseases. Selected records from the above databases will be downloaded to the EndNote X8 for screening independently by two researchers. Two independent reviewers will screen the abstracts of all the articles against the eligibility criteria. The review protocol will be designed and conducted according to the Cochrane Handbook for Systematic Reviews of Interventions^[19]^ and reported complying with the Preferred Reporting Items for Systematic reviews and Meta-Analyses Protocols (PRISMA-P) statement guidelines.^[20]^ A PRISMA flow diagram will be produced to document the whole literature selection process (Fig. 1).

### 2.4. Data extraction

Data extraction will be conducted by two reviewers independently with the software Excel spreadsheet. Extracted data will include important characteristics of the studies including first author, publication year, country, and participants’ demographic characteristics, clinical outcome measures, coaching information and follow up. If there are conflicts regarding an article's inclusion, a third reviewer will be consulted independently. Retained articles will be subject to full-text review.

### 2.5. Quality assessment

The quality of each study will be assessed with the Grading of Recommendations Assessment, Development, and Evaluation (GRADE) by two reviewers independently.^[21]^ The evaluation domains include risk of bias, inconsistency, indirectness, imprecision, and publication bias. Quality of the studies will be judged in “high”, “moderate”, “low” or “very low”. Summary of findings table will be produced. Discrepancies will be resolved through consensus.

### 2.6. Risk of bias assessment

The risk of bias will be evaluated by two reviewers independently with the Cochrane risk of bias tool version 5.1.0.^[22]^ In the event of a discrepancy between the two reviewers, a request will be sent to the original study author for additional information. Each article will be rated as “high”, “unclear” or “low” for performance, attrition, and reporting bias from each of six domains (random sequence generation; allocation concealment; blinding of participants and personnel; blinding of outcome assessment; incomplete outcome data, and selective reporting. The risk of bias summary table will be produced.

For the presence of publication bias, each RCT trial will be double checked for the publication date to make sure all the enrolled studies are previous publications; Study trial outcomes are selectively reported; if sufficient data, a funnel plot will be used to assess the potential for publication bias.

### 2.7. Data synthesis and statistics

Each study result will be synthesized and presented in a narrative form in an Excel file. The results will then be gathered into a summary table. There will be a focus on presenting the descriptive statistics for relevant outcomes. Study characteristics will be summarized with a stand mean difference (SMD) or interquartile range (IQR) for continuous variables, relative risk (RR) for dichotomous outcomes, and frequencies (%) for categorical variables. All the collected RCTs data will be entered into the software Review Manager (Revman version 5.3.5.) provided by the Cochrane Collaboration. A Meta-analysis will be conducted if trials are available and the studies/methods are sufficiently homogeneous regarding the interventions and outcomes from included studies, structured around coaching effect, intervention groups, coaching techniques, outcome measure results and coaching compacity and deliverer. Random-effects models will be used to calculate effect sizes and 95% confidence intervals. The between-study variance will be assessed with the I^2^ index. As far as possible, similar studies will be grouped into subgroups. If a meta-analysis is not conducted for any outcome due to insufficient data, subgroup analyses for comparisons between HC and conventional cares or other alternative therapies will also be conducted. If appropriate, results will be presented by 95% confidence intervals.

## 3 Results

This systematic review is the first study to comprehensively explore the effectiveness and current status of HC intervention for chronic condition patients. This systematic review protocol has been registered on the International Prospective Register of Systematic Reviews (PROSPERO) on April 28, 2020 (Registration number: CRD42020153280) https://www.crd.york.ac.uk/prospero/display_record.php?RecordID=153280.

## 4 Discussion

The rising prevalence of chronic conditions, as well as the significant social and economic burden of these conditions, it is imperative to develop better and innovative strategies to prevent and manage chronic conditions. How to manage the rising prevalence of chronic conditions and the associated costs is the main challenge facing governments and health-care systems.

HC has been increasingly recognized as an Indispensable complement to education-based initiatives for chronic condition patients’ health improvement. It is necessary to conduct a comprehensive overview of the data of the current status of HC on chronic diseases management qualitatively and quantitatively. Our proposed review will synthesize the currently available evidence with rigorous methods, highlight the impact of HC on chronic conditions and overcome the evidence barriers which may impact clinical decision making and guide future research initiatives. The study outcomes will provide strong evidence for patients, health care providers, healthcare systems, public health departments, and insurance companies in considering whether to deliver HC interventions to the patients with chronic conditions. With more supporting materials, HC may become an important guide addressing the health issues and well-being among patients with chronic conditions.

## Acknowledgments

The authors thank the grant support from the HEAD Foundation, Singapore.

## Author contributions

**Conceptualization:** Susanne M. Cutshall, Brent A. Bauer.

**Data curation:** Juan Yang, Stephanie A. Lindeen.

**Formal analysis:** Juan Yang, Manisha Salinas.

**Funding acquisition:** Brent A. Bauer

**Methodology:** Juan Yang, Manisha Salinas

**Project administration:** Brent A. Bauer.

**Resources:** Brent A. Bauer.

**Software:** Juan Yang, Manisha Salinas.

**Supervision:** Brent A. Bauer.

**Writing – original draft:** Juan Yang.

**Writing – review & editing:** Brent A. Bauer, Susanne M. Cutshall, Adam I. Perlman, Abd Moain Abu Dabrh, Kasey R. Boehmer
